# Supplementary material for: Three-Dimensional Cell Culture Models to Investigate Oral Carcinogenesis: A Scoping Review
Source: Int J Mol Sci. 2020 Dec 14;21(24):9520. doi: 10.3390/ijms21249520 (PMC7765087; doi:10.3390/ijms21249520)
Supplement: Supplementary file 1 [file ijms-21-09520-s001.pdf]

Supplementary Materials

Three-Dimensional Cell Culture Models to Investigate Oral Carcinogenesis: A Scoping Review

Table S1. Search strategy in different databases.

Database	Search String
PubMed	((((3d[All Fields] AND ("cell culture techniques"[MeSH Terms] OR ("cell"[All Fields] AND "culture"[All Fields] AND "techniques"[All Fields]) OR "cell culture techniques"[All Fields] OR ("cell"[All Fields] AND "culture"[All Fields]) OR "cell culture"[All Fields])) OR spheroid[All Fields]) OR ("organoids"[MeSH Terms] OR "organoids"[All Fields] OR "organoid"[All Fields])) OR organotypic[All Fields]) AND ((("mouth"[MeSH Terms] OR "mouth"[All Fields] OR "oral"[All Fields]) AND ("carcinoma, squamous cell"[MeSH Terms] OR ("carcinoma"[All Fields] AND "squamous"[All Fields] AND "cell"[All Fields]) OR "squamous cell carcinoma"[All Fields] OR ("squamous"[All Fields] AND "cell"[All Fields] AND "carcinoma"[All Fields]))) OR (("mouth"[MeSH Terms] OR "mouth"[All Fields] OR "oral"[All Fields]) AND ("carcinoma in situ"[MeSH Terms] OR ("carcinoma"[All Fields] AND "situ"[All Fields]) OR "carcinoma in situ"[All Fields] OR ("epithelial"[All Fields] AND "dysplasia"[All Fields]) OR "epithelial dysplasia"[All Fields])))
Web of Science	ts=(spheroid OR organoid OR organotypic) AND ts=(oral squamous cell carcinoma OR oral epithelial dysplasia)<br>Indexes=SCI-EXPANDED, SSCI, A&HCI, CPCI-S, CPCI-SSH, BKCI-S, BKCI-SSH, ESCI, CCR-EXPANDED, IC Timespan=All years
Scopus	(TITLE-ABS-KEY (spheroid) OR TITLE-ABS-KEY (organoid) OR TITLE-ABS-KEY (organotypic) AND TITLE-ABS-KEY (oral AND squamous AND cell AND carcinoma) OR TITLE-ABS-KEY (oral AND epithelial AND dysplasia))

Table S2. List of excluded articles and reasons for exclusion.

S.No	Authors (Year Published)	Reason for Exclusion
1	Burkhardt et al. (1976) [1]	Not associated with 3D cell cultures
2	Schenk (1976) [2]	Not associated with 3D cell cultures
3	Chen et al. (1977)[3]	Not associated with 3D cell cultures
4	Dardick et al. (1984) [4]	Not associated with 3D cell cultures
5	El-labban et al. (1986)[5]	Not associated with 3D cell cultures
6	Sasaki et al. (1988) [6]	Article in Japanese
7	Schwachofer et al. (1989) [7]	Full text not available
8	Croojimans et al. (1990)[8]	Associated with animal cells
9	Park et al. (1995) [9]	Full text not available
10	Aldosari et al. (1996)[10]	Not associated with 3D cell cultures
11	Blant et al. (1996)[11]	Not associated with 3D cell cultures
12	Sacks (1996) [12]	Full text not available
13	Whiteside et al. (1996)[13]	Full text not available
14	Berndt et al. (1998) [14]	Article in German
15	Hanzawa et al. (2000)[15]	Full text not available
16	Berndt et al. (2001)[16]	Full text not available
17	Heimdal et al. (2001)[17]	Full text not available
18	Sandalon et al. (2001) [18]	Cells from the skin epidermis

19	Vondracek et al. (2001)[19]	Only abstract available (conference proceedings)
20	Zhang et al. (2001) [20]	Not associated with 3D cell cultures
21	Yook et al. (2004) [21]	Cells from salivary gland tumour (mucoepidermoid carcinoma)
22	Margulis et al. (2006) [22]	HaCaT cell line from skin epidermis
23	Zhang et al. (2006) [23]	HaCaT cell line from skin epidermis
24	Fischbach et al. (2007) [24]	Associated with animal cells
25	Lin et al. (2007) [25]	Review article
26	Zhang et al. (2007) [26]	Not associated with 3D cell cultures
27	Yoshiba et al. (2009) [27]	Not associated with 3D cell cultures
28	Kang et al. (2010) [28]	Article in Chinese
29	Liang et al. (2010) [29]	Not associated with 3D cell cultures
30	Van Zeeburg et al. (2010) [30]	Primary cells from oro-pharynx
31	Besic Gyenge et al. (2011) [31]	UMB-SCC-745 cell line from Tonsil
32	Hoffmann (2011) [32]	Article in German
33	Lim et al. (2011) [33]	Site vaguely mentioned as 'head and neck'
34	Yang et al. (2011) [34]	Not associated with 3D cell cultures
35	Duarte et al. (2012) [35]	Associated with animal cells
36	Naganuma et al. (2012) [36]	EPC2-hTERT cell line from esophagus
37	Korraah et al. (2012) [37]	Not associated with 3D cell cultures
38	Hisha et al. (2013) [38]	Associated with animal cells
39	Chen et al. (2014) [39]	Associated with animal cells
40	Chen et al. (2014) [40]	Not associated with 3D cell cultures
41	Colley et al. (2014) [41]	FaDu cell line from hypopharynx
42	Maruyama et al. (2014) [42]	Not associated with 3D cell cultures
43	Salo et al. (2015) [43]	Review article
44	Farnebo et al. (2015) [44]	Associated with animal cells
45	Lin et al. (2015) [45]	Cell lines from pharynx and larynx
46	Makielski et al. (2016) [46]	Associated with HPV related oncogenesis
47	Yu and Qui (2016) [47]	HeLa contaminated SCC cell line
48	Bhattacharyya et al (2017) [48]	Associated with explant culture
49	Hanley et al. (2018) [49]	5PT cell line from supraglottis
50	Ihler et al. (2018) [50]	Primary cells from hypopharynx
51	Karakasheva et al. (2018) [51]	TE and HCE cell lines from oesophagus
52	Kaseb et al. (2018) [52] [52]	Vaguely mentioned as 'head and neck'
53	Lee et al. (2018) [53]	Full text not available
54	Matrka et al. (2018) [54]	Associated with animal cells
55	Choi et al. (2019) [55]	FaDu cell line from hypopharynx
56	Guidry et al. (2019) [56]	Associated with viral oncogenesis of oropharyngeal SCC
57	Naakka et al. (2019) [57]	UT-SCC-42B cell line from larynx
58	Oak et al. (2019) [58]	Only abstract available (conference proceedings)
59	Olek et al. (2019) [59]	Review article

References

1. Burkhardt, A.; Bommer, G.; Gebbers, J.O.; Höltje, W.J. Formation of giant cells in oral squamous cell carcinoma during bleomycin treatment—Enzymehistochemical, electronmicroscopic and ultrahistochemical investigations. *Virchows Archiv A Pathological Anatomy and Histology* **1976**, *369*, 197–214, doi:10.1007/bf00427709.
2. Schenk, P. The ultrastructure of dyskeratotic and dysplastic keratinocytes in oral epithelium. *HNO* **1976**, *24*, 147–160.

3. Chen, S.Y.; Harwick, R.D. Ultrastructure of oral squamous-cell carcinoma. *Oral Surgery, Oral Medicine, Oral Pathology* **1977**, *44*, 744–753, doi:10.1016/0030-4220(77)90384-x.
4. Dardick, I.; Daya, D.; Hardie, J.; van Nostrand, A.W. Mucoepidermoid carcinoma: Ultrastructural and histogenetic aspects. *J Oral Pathol* **1984**, *13*, 342–358.
5. el-Labban, N.G.; Osorio-Herrera, E. Apoptotic bodies and abnormally dividing epithelial cells in squamous cell carcinoma. *Histopathology* **1986**, *10*, 921–931, doi:10.1111/j.1365-2559.1986.tb02590.x [doi].
6. Sasaki, T.; Sugiyama, S.; Kuwahara, K.; Kitahara, M.; Mashiyama, S.; Takahashi, K.; Kanno, S. [Biological basis for combined radio-chemotherapy in radioresistant tumors]. *Gan No Rinsho* **1988**, *34*, 1877–1886.
7. Schwachofer, J.H.; Crooijmans, R.P.; van Gasteren, J.J.; Hoogenhout, J.; Jerusalem, C.R.; Kal, H.B.; Theeuwes, A.G. Radiosensitivity of different human tumor cells lines grown as multicellular spheroids determined from growth curves and survival data. *Int J Radiat Oncol Biol Phys* **1989**, *17*, 1015–1020, doi10.1016/0360-3016(89)90149-1.
8. Crooijmans, R.P.M.A.; Schwachöfer, J.H.M.; Hoogenhout, J.; Merkx, G.; Poels, L.G.; Jap, P.H.K.; Ramaekers, F.C.S.; Mijnheere, E.P.; Elprana, D.; Thomas, C.M.G. Cell lines of human oral squamous-cell carcinomas retaining their differentiated phenotype. *International Journal of Cancer* **1990**, *45*, 945–951, doi:10.1002/ijc.2910450528.
9. Park, N.H.; Gujuluva, C.N.; Baek, J.H.; Cherrick, H.M.; Shin, K.H.; Min, B.M. Combined oral carcinogenicity of HPV-16 and benzo(a)pyrene: An in vitro multistep carcinogenesis model. *Oncogene* **1995**, *10*, 2145–2153.
10. Aldosari, A.; McDonald, J.; Olson, B.; Noblitt, T.; Li, Y.M.; Stookey, G. Influence of benzylisothiocyanate and 13-cis-retinoic acid on micronucleus formation induced by benzo[a]pyrene. *Mutat. Res.-Fundam. Mol. Mech. Mutagen.* **1996**, *352*, 1–7, doi:10.1016/0027-5107(96)00149-2.
11. Blant, S.A.; Woodtli, A.; Wagnieres, G.; Fontolliet, C.; vandenBergh, H.; Monnier, P. In vivo fluence rate effect in photodynamic therapy of early cancers with tetra(m-hydroxyphenyl)chlorin. *Photochem. Photobiol.* **1996**, *64*, 963–968, doi:10.1111/j.1751-1097.1996.tb01862.x.
12. Sacks, P.G. Cell, tissue and organ culture as in vitro models to study the biology of squamous cell carcinomas of the head and neck. *Cancer Metastasis Rev.* **1996**, *15*, 27–51, doi:10.1007/bf00049486.
13. Whiteside, T.L.; Chikamatsu, K.; Nagashima, S.; Okada, K. Antitumor effects of cytolytic T lymphocytes (CTL) and natural killer (NK) cells in head and neck cancer. *Anticancer Res.* **1996**, *16*, 2357–2364.
14. Berndt, A.; Hyckel, P.; Konneker, A.; Kosmehl, H. [3-dimensional in vitro invasion model for oral squamous epithelial carcinomas. Evaluation of tumor and stromal cell properties as well as extracellular matrix]. *Mund Kiefer Gesichtschir* **1998**, *2*, 256–260, doi:10.1007/s100060050070.
15. Hanzawa, M.; Shindoh, M.; Higashino, F.; Yasuda, M.; Inoue, N.; Hida, K.; Ono, M.; Kohgo, T.; Nakamura, M.; Notani, K.I.; et al. Hepatocyte growth factor upregulates E1AF that induces oral squamous cell carcinoma cell invasion by activating matrix metalloproteinase genes. *Carcinogenesis* **2000**, *21*, 1079–1085.
16. Berndt, A.; Borsi, L.; Hyckel, P.; Kosmehl, H. Fibrillary co-deposition of laminin-5 and large unspliced tenascin-C in the invasive front of oral squamous cell carcinoma in vivo and in vitro. *Journal of Cancer Research and Clinical Oncology, Supplement* **2001**, *127*, 286–292.
17. Heimdal, J.H.; Olsnes, C.; Olofsson, J.; Aarstad, H.J. Monocyte and monocyte-derived macrophage secretion of MCP-1 in co-culture with autologous malignant and benign control fragment spheroids. *Cancer Immunol Immunother* **2001**, *50*, 300–306.
18. Sandalon, Z.; Fusenig, N.E.; McCutcheon, J.; Taichman, L.B.; Garlick, J.A. Suicide gene therapy for premalignant disease: A new strategy for the treatment of intraepithelial neoplasia. *Gene Ther* **2001**, *8*, 232–238, doi:10.1038/sj.gt.3301344.
19. Vondracek, M.; Hansson, A.; Grafstom, R. Assessment of keratin expression and xenobiotic metabolism in cultured normal and transformed human oral keratinocytes; Springer: Dordrecht, 2001; Vol. 1, pp. 143-148.
20. Zhang, J.C.; Savage, H.E.; Sacks, P.G.; Delohery, T.; Alfano, R.R.; Katz, A.; Schantz, S.P. Innate cellular fluorescence reflects alterations in cellular proliferation. *Lasers Surg. Med.* **1997**, *20*, 319–331, doi:10.1002/(sici)1096-9101(1997)20:3<319::Aid-lsm11>3.0.Co;2-8.
21. Yook, J.I.; Lee, S.A.; Chun, Y.C.; Huh, J.; Cha, I.H.; Kim, J. The myoepithelial cell differentiation of mucoepidermoid carcinoma in a collagen gel-based coculture model. *J Oral Pathol Med* **2004**, *33*, 237–242, doi:10.1111/j.0904-2512.2004.00056.x.
22. Margulis, A.; Zhang, W.; Alt-Holland, A.; Pawagi, S.; Prabhu, P.; Cao, J.; Zucker, S.; Pfeiffer, L.; Garfield, J.; Fusenig, N.E.; et al. Loss of intercellular adhesion activates a transition from low- to high-grade human squamous cell carcinoma. *Int J Cancer* **2006**, *118*, 821–831, doi:10.1002/ijc.21409.

23. Zhang, W.; Alt-Holland, A.; Margulis, A.; Shamis, Y.; Fusenig, N.E.; Rodeck, U.; Garlick, J.A. E-cadherin loss promotes the initiation of squamous cell carcinoma invasion through modulation of integrin-mediated adhesion. *J Cell Sci* **2006**, *119*, 283–291, doi:10.1242/jcs.02738.
24. Fischbach, C.; Chen, R.; Matsumoto, T.; Schmelzle, T.; Brugge, J.S.; Polverini, P.J.; Mooney, D.J. Engineering tumors with 3D scaffolds. *Nat Methods* **2007**, *4*, 855–860, doi:10.1038/nmeth1085.
25. Lin, C.J.; Grandis, J.R.; Carey, T.E.; Gollin, S.M.; Whiteside, T.L.; Koch, W.M.; Ferris, R.L.; Lai, S.Y. Head and neck squamous cell carcinoma cell lines: Established models and rationale for selection. *Head Neck-J. Sci. Spec. Head Neck* **2007**, *29*, 163–188, doi:10.1002/hed.20478.
26. Zhang, X.; Chen, Z.; Khuri, F.R.; Shin, D.M. Induction of cell cycle arrest and apoptosis by a combined treatment with 13-cis-retinoic acid, interferon-alpha 2a, and alpha-tocopherol in squamous cell carcinoma of the head and neck. *Head Neck-J. Sci. Spec. Head Neck* **2007**, *29*, 351–361, doi:10.1002/hed.20525.
27. Yoshiba, S.; Ito, D.; Nagumo, T.; Shirota, T.; Hatori, M.; Shintani, S. Hypoxia induces resistance to 5-fluorouracil in oral cancer cells via G(1) phase cell cycle arrest. *Oral Oncology* **2009**, *45*, 109–115, doi:10.1016/j.oraloncology.2008.04.002.
28. Kang, F.W.; Wang, K.; Wu, M.; Wang, Z.L.; Zhu, Y.; Min, R. [Biological characteristics of CD133+ subpopulation in tongue squamous cell carcinoma Tca8113 cell line]. *Hua Xi Kou Qiang Yi Xue Za Zhi* **2010**, *28*, 560–564.
29. Liang, C.H.; Wang, G.H.; Hung, W.J.; Lin, R.J.; Cheng, D.L.; Chou, T.H. Apoptosis effect of Sinularia leptoclados, S. depressan and S. inflate extracts in human oral squamous cell carcinomas. *J. Taiwan Inst. Chem. Eng.* **2010**, *41*, 86–91, doi:10.1016/j.jtice.2009.05.014.
30. Van Zeeburg, H.J.T.; Van Beusechem, V.W.; Huizenga, A.; Haisma, H.J.; Korokhov, N.; Gibbs, S.; René Leemans, C.; Brakenhoff, R.H. Adenovirus retargeting to surface expressed antigens on oral mucosa. *J. Gene. Med.* **2010**, *12*, 365–376, doi:10.1002/jgm.1447.
31. Besic Gyenge, E.; Darphin, X.; Wirth, A.; Pieles, U.; Walt, H.; Bredell, M.; Maake, C. Uptake and fate of surface modified silica nanoparticles in head and neck squamous cell carcinoma. *J Nanobiotechnology* **2011**, *9*, 32, doi:10.1186/1477-3155-9-32.
32. Hoffmann, T.K. Immunotherapy of head and neck cancer. *Hno* **2011**, *59*, 224–229, doi:10.1007/s00106-010-2251-2.
33. Lim, Y.C.; Oh, S.Y.; Cha, Y.Y.; Kim, S.H.; Jin, X.; Kim, H. Cancer stem cell traits in squamospheres derived from primary head and neck squamous cell carcinomas. *Oral Oncology* **2011**, *47*, 83–91, doi:10.1016/j.oraloncology.2010.11.011.
34. Yang, S.; Chen, S.; Chen, X.; Long, X. Primary typical carcinoid tumour in the retromolar region with prominent squamous differentiation: A case report. *Int. J. Oral Maxillofac. Surg.* **2011**, *40*, 991–994, doi:10.1016/j.ijom.2011.03.008.
35. Duarte, S.; Loubat, A.; Momier, D.; Topi, M.; Faneca, H.; Pedroso De Lima, M.C.; Carle, G.F.; Pierrefite-Carle, V. Isolation of head and neck squamous carcinoma cancer stem-like cells in a syngeneic mouse model and analysis of hypoxia effect. *Oncology Reports* **2012**, *28*, 1057–1062, doi:10.3892/or.2012.1904.
36. Naganuma, S.; Whelan, K.A.; Natsuizaka, M.; Kagawa, S.; Kinugasa, H.; Chang, S.; Subramanian, H.; Rhoades, B.; Ohashi, S.; Itoh, H.; et al. Notch receptor inhibition reveals the importance of cyclin D1 and Wnt signaling in invasive esophageal squamous cell carcinoma. *Am. J. Cancer Res.* **2012**, *2*, 459–475.
37. Korraah, A.; Odenthal, M.; Kopp, M.; Vigneswaran, N.; Sacks, P.G.; Dienes, H.P.; Stutzer, H.; Niedermeier, W. Induction of apoptosis and up-regulation of cellular proliferation in oral leukoplakia cell lines inside electric field. *Oral Surg. Oral Med. Oral Pathol. Oral Radiol.* **2012**, *113*, 644–654, doi:10.1016/j.oooo.2011.11.016.
38. Hisha, H.; Tanaka, T.; Kanno, S.; Tokuyama, Y.; Komai, Y.; Ohe, S.; Yanai, H.; Omachi, T.; Ueno, H. Establishment of a novel lingual organoid culture system: Generation of organoids having mature keratinized epithelium from adult epithelial stem cells. *Scientific reports* **2013**, *3*, 3224–3224, doi:10.1038/srep03224.
39. Chen, Y.K.; Huang, A.H.C.; Lin, L.M. Sphere-forming-like cells (squamospheres) with cancer stem-like cell traits from VX2 rabbit buccal squamous cell carcinoma. *International Journal of Oral Science* **2014**, *6*, 212–218, doi:10.1038/ijos.2014.32.
40. Chen, Y.; Yan, W.X.; He, S.Q.; Chen, J.C.; Chen, D.; Zhang, Z.Q.; Liu, Z.G.; Ding, X.Q.; Wang, A.X. In vitro effect of iASPP on cell growth of oral tongue squamous cell carcinoma. *Chin. J. Cancer Res.* **2014**, *26*, 382–390, doi:10.3978/j.issn.1000-9604.2014.07.05.
41. Colley, H.E.; Hearnden, V.; Avila-Olias, M.; Cecchin, D.; Canton, I.; Madsen, J.; Macneil, S.; Warren, N.; Hu, K.; McKeating, J.A.; et al. Polymersome-mediated delivery of combination anticancer therapy to head and

neck cancer cells: 2D and 3D in vitro evaluation. *Molecular Pharmaceutics* **2014**, *11*, 1176–1188, doi:10.1021/mp400610b.

42. Maruyama, S.; Shimazu, Y.; Kudo, T.; Sato, K.; Yamazaki, M.; Abe, T.; Babkair, H.; Cheng, J.; Aoba, T.; Saku, T. Three-dimensional visualization of perlecan-rich neoplastic stroma induced concurrently with the invasion of oral squamous cell carcinoma. *J Oral Pathol Med* **2014**, *43*, 627–636, doi:10.1111/jop.12184 [doi].
43. Salo, T.; Vered, M.; Bello, I.O.; Nyberg, P.; Bitu, C.C.; Hurvitz, A.Z.; Dayan, D. Insights into the role of components of the tumor microenvironment in oral carcinoma call for new therapeutic approaches. *Experimental Cell Research* **2014**, *325*, 58–64, doi:10.1016/j.yexcr.2013.12.029.
44. Farnebo, L.; Shahangian, A.; Lee, Y.; Shin, J.H.; Scheeren, F.A.; Sunwoo, J.B. Targeting Toll-like receptor 2 inhibits growth of head and neck squamous cell carcinoma. *Oncotarget* **2015**, *6*, 9897–9907, doi:10.18632/oncotarget.3393.
45. Lin, Y.; Mallen-St Clair, J.; Luo, J.; Sharma, S.; Dubinett, S.; St John, M. p53 modulates NF-kappa B mediated epithelial-to-mesenchymal transition in head and neck squamous cell carcinoma. *Oral Oncology* **2015**, *51*, 921–928, doi:10.1016/j.oraloncology.2015.07.006.
46. Makielski, K.R.; Lee, D.; Lorenz, L.D.; Nawandar, D.M.; Chiu, Y.F.; Kenney, S.C.; Lambert, P.F. Human papillomavirus promotes Epstein-Barr virus maintenance and lytic reactivation in immortalized oral keratinocytes. *Virology* **2016**, *495*, 52–62, doi:10.1016/j.virol.2016.05.005.
47. Yu, Y.; Qiu, L. Optimizing particle size of docetaxel-loaded micelles for enhanced treatment of oral epidermoid carcinoma. *Nanomedicine: Nanotechnology, Biology, and Medicine* **2016**, *12*, 1941–1949, doi:10.1016/j.nano.2016.04.012.
48. Bhattacharyya, S.; Sekar, V.; Majumder, B.; Mehrotra, D.G.; Banerjee, S.; Bhowmick, A.K.; Alam, N.; Mandal, G.K.; Biswas, J.; Majumder, P.K.; et al. CDKN2A-p53 mediated antitumor effect of Lupeol in head and neck cancer. *Cell Oncol (Dordr)* **2017**, *40*, 145–155, doi:10.1007/s13402-016-0311-7..
49. Hanley, C.J.; Mellone, M.; Ford, K.; Thirdborough, S.M.; Mellows, T.; Frampton, S.J.; Smith, D.M.; Harden, E.; Szyndralewiez, C.; Bullock, M.; et al. Targeting the Myofibroblastic Cancer-Associated Fibroblast Phenotype Through Inhibition of NOX4. *Journal of the National Cancer Institute* **2018**, *110*, doi:10.1093/jnci/djx121.
50. Ihler, F.; Gratz, R.; Wolff, H.A.; Weiss, B.G.; Bertlich, M.; Kitz, J.; Salinas, G.; Rave-Frank, M.; Canis, M. Epithelial-Mesenchymal Transition during Metastasis of HPV-Negative Pharyngeal Squamous Cell Carcinoma. *Biomed Research International* **2018**, 7929104.doi:10.1155/2018/7929104.
51. Karakasheva, T.A.; Lin, E.W.; Tang, Q.S.; Qiao, E.; Waldron, T.J.; Soni, M.; Klein-Szanto, A.J.; Sahu, V.; Basu, D.; Ohashi, S.; et al. IL-6 Mediates Cross-Talk between Tumor Cells and Activated Fibroblasts in the Tumor Microenvironment. *Cancer Research* **2018**, *78*, 4957–4970, doi:10.1158/0008-5472.Can-17-2268.
52. Kaseb, H.O.; Fohrer-Ting, H.; Lewis, D.W.; Lagasse, E.; Gollin, S.M. Identification, expansion and characterization of cancer cells with stem cell properties from head and neck squamous cell carcinomas. *Experimental Cell Research* **2016**, *348*, 75–86, doi:10.1016/j.yexcr.2016.09.003.
53. Lee, M.K.; Park, J.H.; Gi, S.H.; Hwang, Y.S. IL-1beta Induces Fascin Expression and Increases Cancer Invasion. *Anticancer Res* **2018**, *38*, 6127–6132, doi:10.21873/anticanres.12964.
54. Matrka, M.C.; Cimperman, K.A.; Haas, S.R.; Guasch, G.; Ehrman, L.A.; Waclaw, R.R.; Komurov, K.; Lane, A.; Wikenheiser-Brokamp, K.A.; Wells, S.I. Dek overexpression in murine epithelia increases overt esophageal squamous cell carcinoma incidence. *PLoS Genet.* **2018**, *14*, doi:10.1371/journal.pgen.1007227.
55. Choi, S.Y.; Oh, S.Y.; Kang, S.H.; Kang, S.M.; Kim, J.; Lee, H.J.; Kwon, T.G.; Kim, J.W.; Hong, S.H. NAB 2-Expressing Cancer-Associated Fibroblast Promotes HNSCC Progression. *Cancers (Basel)* **2019**, *11*, doi:10.3390/cancers11030388.
56. Guidry, J.T.; Myers, J.E.; Bienkowska-Haba, M.; Songock, W.K.; Ma, X.; Shi, M.; Nathan, C.O.; Bodily, J.M.; Sapp, M.J.; Scott, R.S. Inhibition of Epstein-Barr virus replication in human papillomavirus-immortalized keratinocytes. *Journal of Virology* **2019**, *93*, doi:10.1128/jvi.01216-18.
57. Naakka, E.; Tuomainen, K.; Wistrand, H.; Palkama, M.; Suleymanova, I.; Al-Samadi, A.; Salo, T. Fully Human Tumor-based Matrix in Three-dimensional Spheroid Invasion Assay. *J Vis Exp* **2019**. doi:10.3791/59567.
58. Oak, A.S.; Kim, T.; Bocheva, G.; Elsayed, M.; Brozyna, A.A.; Janjetovic, Z.; Athar, M.; Slominski, A.T. Novel noncalcemic vitamin D hydroxyderivatives downregulate SHH and Wnt signaling pathways and inhibit spheroid formation in human oral squamous cell carcinoma and murine basal cell carcinoma. *J. Invest. Dermatol.* **2019**, *139*, B5–B5, doi:10.1016/j.jid.2019.06.021.

59. Olek, M.; Kasperski, J.; Skaba, D.; Wiench, R.; Cieślar, G.; Kawczyk-Krupka, A. Photodynamic therapy for the treatment of oral squamous carcinoma—Clinical implications resulting from in vitro research. *Photodiagnosis and Photodynamic Therapy* **2019**, *27*, 255–267, doi:10.1016/j.pdpdt.2019.06.012.

Table S3. Studies utilizing spheroids in OSCC.

Study No.	Authors (Year Published)	Method of Spheroid Generation	Type of Cell Used and Site from Which Cells Were Derived	Whether Co-Cultured
1	Heimdal et al., (2001) [1]	Agar coated tissue culture flasks.	Primary OSCC cells—oral cavity.	Yes—monocytes
2	Harper et al., (2007) [2]	10 cm dishes pre-coated with Poly-HEMA.	Cancer cell line (H357)—tongue.	No
3	Rasanen et al., (2009) [3]	6 well plates coated with 0.6% agarose.	Cancer cell lines (SCC9, SCC25)—tongue.	No
4	Tsai et al., (2011) [4]	Not mentioned.	Cancer cell line (OC2)—buccal mucosa.	No
5	Chen et al., (2012) [5]	10 cm dish coated with agarose.	Cancer cell line (SCC25)—tongue.	No
6	Chen et al., (2012) [6]	Agarose coated 10 cm dish.	Cancer cell lines (SAS—tongue, OECM1—Oral Cavity).	No
7	Chen et al., (2012) [7]	10 mm dishes.	Cancer cell lines (SAS—Tongue, OECM1—oral cavity).	No
8	Dennis et al., (2012) [8]	Cells embedded within the matrix method using growth factor reduced Matrigel.	Normal oral keratinocyte cell lines (OKF-6—floor of the mouth, HOK—oral cavity). Cancer cell lines (Tu686- larynx Tu212- head and neck).	No
9	Chang et al., (2013) [9]	Not mentioned.	Cancer cell line (OECM1—oral cavity).	No
10	Deng et al., (2013) [10]	Commercially available ULA 10-cm plate.	Cancer cell line (Cal27—tongue).	No
11	Huang et al., (2014) [11]	Commercially available 6 well ULA plate.	Cancer cell lines (Ca9-22—gingiva, SAS, SCC4—tongue).	No
12	Lee et al., (2014) [12]	0.6% agarose coated plate.	Cancer cell line (SCC9-tongue).	No
13	Ayuso et al., (2015) [13]	Spheroids first generated by hanging drop method then transferred to collagen and into a microfluidic device.	Cancer cell line (OSC19—tongue SCC metastasized to cervical lymph node).	No
14	Bedal et al., (2015) [14]	Cells above the matrix method using Matrigel.	Cancer cell line (PCI13—oral cavity).	No
15	Chou et al., (2015) [15]	Commercially available 6 well ULA plates.	Cancer cell lines (SAS—tongue OECM1—oral cavity).	No
16	Desiderio et al.,(2015) [16]	Commercially available ULA flasks.	Cancer cell lines (SCC14B—floor of the mouth, SCC103—tongue).	No

17	Kadletz et al., (2015) [17]	Commercially available 96 well ULA plates.	Cancer cell lines (Cal27, SCC25—tongue).	No
18	Lin et al., (2015) [18]	Commercially available 6 well ULA plates.	Cancer cell lines (SAS, HSC3, TW2.6 and SCC4—tongue).	No
19	Ma et al., (2015) [19]	Commercially available 6 well ULA plates.	Cancer cell line (Cal27—tongue).	No
20	Hinger et al., (2016) [20]	Spheroids first generated by hanging drop method and transferred to 96 well plates coated with 1.5% agarose.	Cancer cell line (Cal 33—tongue).	No
21	Lee and Ramos (2016) [21]	0.6% agarose coated tissue culture plate.	Cancer cell line (SCC9—tongue).	No
22	Lee et al., (2016) [22]	0.6% agarose coated plate.	Cancer cell line (SCC9—tongue).	No
23	Lee et al., (2016) [23]	Commercially available 6 well ULA plates.	Cancer cell lines (SCC9—tongue, HOK-16B BapT—carcinogen transformed human oral keratinocyte).	No
24	Moon et al., (2016) [24]	Non-adherent 24-well culture plates coated with a 10 % poly HEMA.	Cancer cell line (YD-10B—tongue).	No
25	Patel and Rakesh (2016) [25]	Commercially available 6 well ULA plates.	Primary OSCC cells (buccal mucosa and tongue).	No
26	de Campos et al., (2017) [26]	Agarose coated 96 well plate.	Cancer cell lines (CAL27, and SCC25—tongue).	No
27	Hageman et al., (2017) [27]	Hanging drop and commercially available ULA plates.	Cancer cell line (CAL27—tongue).	No
28	Leong et al., (2017) [28]	Aerosol based microencapsulation technique.	Cancer cell line (ORL-48—gingiva).	No
29	Mendonca et al., (2017) [29]	U wells coated with 1% agarose.	Cancer cell line (SCC9—tongue).	No
30	Mohanta et al.,(2017) [30]	24 well ultra-low attachment plates.	Cancer cell lines (SCC029B, AW13516, SCC040, CAL-27, SCC103 and SCC16—oral cavity).	No
31	O'Rourke et al., (2017) [31]	Cells embedded within the matrix method using rat collagen—type 1.	Cancer cell line (PC130—tongue).	No
32	Ong et al., (2017) [32]	3D printed microfluidic perfusion device.	Cancer cell line (HN137- oral cavity SCC metastasized to cervical lymph node).	No
33	Bano et al., (2018) [33]	Non-adherent 6 well cell culture plates treated with poly-HEMA.	Cancer cell line (SCC-131—floor of mouth).	No

34	Essid et al., (2018) [34]	Non-adherent cell culture plates treated with poly-HEMA.	Cancer cell line (Cal 33—tongue).	No
35	Fujibayashi et al., (2018) [35]	Spheroid catch system.	Cancer cell line (SAS—tongue).	No
36	Guerrriero et al., (2018) [36]	CELLine AD1000 flask system.	Cancer cell line (PE/CA-PJ49/E10—oral cavity).	No
37	Hoornstra et al., (2018) [37]	Spheroid first generated by commercially available 96 well ULA followed by addition of myogel and collagen to the well.	Cancer cell lines (HSC-3, SCC-25—tongue).	No
38	Lazarevic et al., (2018) [38]	Commercially available 24 ULA plates.	Primary OSCC cells (tongue and floor of mouth).	No
39	Noi et al., (2018) [39]	Cells above the matrix method using non-woven silica fibre sheet.	Cancer cell lines (HSC 3- tongue SCC metastasized to cervical lymph node, HSC4—tongue).	No
40	Rivera et al., (2018) [40]	150 mm dish coated with 3.2% agarose.	Cancer cell lines (HSC-3, SCC-9—tongue).	No
41	Roy et al., (2018) [41] [41]	96 well-plated coated with 1% agarose.	Cancer cell line (SCC 131—floor of the mouth).	No
42	Saha et al., (2018) [42]	12 well plates coated with 1% agarose.	Cancer cell lines (Cal 27—tongue SCC 131—floor of mouth).	No
43	Sievers et al., (2018) [43]	96 well-plated coated with 1.5% agarose.	Cancer cell lines (BHY- mandibular alveolus).	No
44	Xie et al., (2018) [44]	Commercially available ULA 6 well plate.	Cancer cell lines (CAL27 and SCC9—tongue).	No
45	Almahmoudi et al., (2019) [45]	Spheroid first generated by commercially available 96 well ULA plates followed by addition of myogel and rat tail collagen 1 to the well.	Cancer cell lines (HSC 3- tongue SCC metastasized to cervical lymph node, SCC 25—tongue).	Yes—cancer-associated fibroblasts
46	Chen et al., (2019) [46]	Cells embedded within the matrix method using Matrigel.	Cancer cell lines (SCC-4, SCC-9, SCC-15, SCC-25 and CAL 27—tongue).	No
47	Furqan et al., (2019) [47]	Cells embedded within the matrix method using Matrigel.	Cancer cell line (ORL-15—oral cavity ORL 48—gingiva).	No
48	Gawas et al., (2019) [48]	Commercially available 6 well ULA plates.	Cancer cell lines (ACOSC3, ACOSC4 and ACOSC16—buccal mucosa).	No
49	Kochanek Stanton et al., (2019) [49]	Commercially available 384 well ULA plates.	Cancer cell lines (UMSCC1, SCC9, PCI-13, Cal33, Cal27, and BICR56—oral cavity, OSC-	No

			19—tongue SCC metastasized to cervical lymph node).	
50	Lang et al., (2019) [50]	Cells above the matrix method using commercially available SeedEZTM scaffold pre-coated with Poly-D-Lysine solution.	Cancer cell lines (HN6, HN8, HN12, HN13, and HN17—tongue).	No
51	Roh et al., (2019) [51]	Cells embedded within the matrix method using Type 1 collagen.	Cancer cell line (Cal27—tongue).	No
52	Su et al., (2019) [52]	ULA multiwell plate.	Cancer cell line (Cal27—tongue).	No
53	Wessely et al., (2019) [53]	Spheroids first formed using hanging drop method and then transferred to Boyden chamber lined by Matrigel.	Cancer cell lines (PCI9—tongue base PCI13—retromolar triangle, PCI68—tongue).	Yes—bone marrow derived stem cells

HEMA—Hydroxyethylmethacrylate, OSCC—Oral squamous cell carcinoma, ULA—Ultra low-attachment, SCC—Squamous cell carcinoma.

References

1. Heimdal, J.H.; Aarstad, H.J.; Olsnes, C.; Olofsson, J. Human autologous monocytes and monocyte-derived macrophages in co-culture with carcinoma F-spheroids secrete IL-6 by a non-CD14-dependent pathway. *Scand J Immunol* **2001**, *53*, 162–170, doi:10.1046/j.1365-3083.2001.00853.x.
2. Harper, L.J.; Piper, K.; Common, J.; Fortune, F.; Mackenzie, I.C. Stem cell patterns in cell lines derived from head and neck squamous cell carcinoma. *Journal of Oral Pathology and Medicine* **2007**, *36*, 594–603, doi:10.1111/j.1600-0714.2007.00617.x.
3. Räsänen, K.; Virtanen, I.; Salmenperä, P.; Grenman, R.; Vaheri, A. Differences in the nemosis response of normal and cancer-associated fibroblasts from patients with oral squamous cell carcinoma. *PLoS ONE* **2009**, *4*, doi:10.1371/journal.pone.0006879.
4. Tsai, L.L.; Yu, C.C.; Chang, Y.C.; Yu, C.H.; Chou, M.Y. Markedly increased Oct4 and Nanog expression correlates with cisplatin resistance in oral squamous cell carcinoma. *Journal of Oral Pathology and Medicine* **2011**, *40*, 621–628, doi:10.1111/j.1600-0714.2011.01015.x.
5. Chen, S.F.; Nieh, S.; Jao, S.W.; Liu, C.L.; Wu, C.H.; Chang, Y.C.; Yang, C.Y.; Lin, Y.S. Quercetin Suppresses Drug-Resistant Spheres via the p38 MAPK-Hsp27 Apoptotic Pathway in Oral Cancer Cells. *PLoS ONE* **2012**, *7*, doi:10.1371/journal.pone.0049275.
6. Chen, S.F.; Chang, Y.C.; Nieh, S.; Liu, C.L.; Yang, C.Y.; Lin, Y.S. Nonadhesive culture system as a model of rapid sphere formation with cancer stem cell properties. *PLoS ONE* **2012**, *7*, doi:10.1371/journal.pone.0031864.
7. Chen, Y.S.; Wu, M.J.; Huang, C.Y.; Lin, S.C.; Chuang, T.H.; Yu, C.C.; Lo, J.F. CD133/Src axis mediates tumor initiating property and epithelial-mesenchymal transition of head and neck cancer. *PLoS ONE* **2011**, *6*, e28053, doi:10.1371/journal.pone.0028053.
8. Dennis, M.; Wang, G.Y.; Luo, J.; Lin, Y.; Dohadwala, M.; Abemayor, E.; Elashoff, D.A.; Sharma, S.; Dubinett, S.M.; St John, M.A. Snail Controls the Mesenchymal Phenotype and Drives Erlotinib Resistance in Oral Epithelial and Head and Neck Squamous Cell Carcinoma Cells. *Otolaryngol. Head Neck Surg.* **2012**, *147*, 726–732, doi:10.1177/0194599812446407.
9. Chang, C.C.; Hsu, W.H.; Wang, C.C.; Chou, C.H.; Kuo, M.Y.; Lin, B.R.; Chen, S.T.; Tai, S.K.; Kuo, M.L.; Yang, M.H. Connective tissue growth factor activates pluripotency genes and mesenchymal-epithelial transition in head and neck cancer cells. *Cancer Res* **2013**, *73*, 4147–4157, doi:10.1158/0008-5472.CAN-12-4085.
10. Deng, R.; Wang, X.; Liu, Y.; Yan, M.; Hanada, S.; Xu, Q.; Zhang, J.; Han, Z.; Chen, W.; Zhang, P. A new gamboge derivative compound 2 inhibits cancer stem-like cells via suppressing EGFR tyrosine phosphorylation in head and neck squamous cell carcinoma. *J Cell Mol Med* **2013**, *17*, 1422–1433, doi:10.1111/jcmm.12129.
11. Huang, C.E.; Yu, C.C.; Hu, F.W.; Chou, M.Y.; Tsai, L.L. Enhanced Chemosensitivity by Targeting Nanog in Head and Neck Squamous Cell Carcinomas. *International Journal of Molecular Sciences* **2014**, *15*, 14935–14948, doi:10.3390/ijms150914935.
12. Lee, C.; Lee, C.; Atakilit, A.; Siu, A.; Ramos, D.M. Differential spheroid formation by oral cancer cells. *Anticancer Res.* **2014**, *34*, 6945–6949.
13. Ayuso, J.M.; Basheer, H.A.; Monge, R.; Sánchez-Álvarez, P.; Doblaré, M.; Shnyder, S.D.; Vinader, V.; Afarinkia, K.; Fernández, L.J.; Ochoa, I. Study of the chemotactic response of multicellular spheroids in a microfluidic device. *PLoS ONE* **2015**, *10*, doi:10.1371/journal.pone.0139515.
14. Bedal, K.B.; Grassel, S.; Spanier, G.; Reichert, T.E.; Bauer, R.J. The NC11 domain of human collagen XVI induces vasculogenic mimicry in oral squamous cell carcinoma cells. *Carcinogenesis* **2015**, *36*, 1429–1439, doi:10.1093/carcin/bgv141.
15. Chou, M.Y.; Hu, F.W.; Yu, C.H.; Yu, C.C. Sox2 expression involvement in the oncogenicity and radiochemoresistance of oral cancer stem cells. *Oral Oncology* **2015**, *51*, 31–39, doi:10.1016/j.oraloncology.2014.10.002.
16. Desiderio, V.; Papagerakis, P.; Tirino, V.; Zheng, L.; Matossian, M.; Prince, M.E.; Paino, F.; Mele, L.; Papaccio, F.; Montella, R.; et al. Increased fucosylation has a pivotal role in invasive and metastatic properties of head and neck cancer stem cells. *Oncotarget* **2015**, *6*, 71–84, doi:10.18632/oncotarget.2698.
17. Kadletz, L.; Heiduschka, G.; Domayer, J.; Schmid, R.; Enzenhofer, E.; Thurnher, D. Evaluation of spheroid head and neck squamous cell carcinoma cell models in comparison to monolayer cultures. *Oncol Lett* **2015**, *10*, 1281–1286, doi:10.3892/ol.2015.3487.

18. Lin, C.S.; Lin, Y.C.; Adebayo, B.O.; Wu, A.; Chen, J.H.; Peng, Y.J.; Cheng, M.F.; Lee, W.H.; Hsiao, M.; Chao, T.Y.; et al. Silencing JARID1B suppresses oncogenicity, stemness and increases radiation sensitivity in human oral carcinoma. *Cancer letters* **2015**, *368*, 36–45, doi:10.1016/j.canlet.2015.07.003.
19. Ma, S.R.; Wang, W.M.; Huang, C.F.; Zhang, W.F.; Sun, Z.J. Anterior gradient protein 2 expression in high grade head and neck squamous cell carcinoma correlated with cancer stem cell and epithelial mesenchymal transition. *Oncotarget* **2015**, *6*, 8807–8821, doi:10.18632/oncotarget.3556.
20. Hinger, D.; Navarro, F.; Käch, A.; Thomann, J.-S.; Mittler, F.; Couffin, A.-C.; Maake, C. Photoinduced effects of m-tetrahydroxyphenylchlorin loaded lipid nanoemulsions on multicellular tumor spheroids. *Journal of Nanobiotechnology* **2016**, *14*, 68, doi:10.1186/s12951-016-0221-x.
21. Lee, C.; Ramos, D.M. Regulation of multicellular spheroids by MAPK and FYN kinase. *Anticancer Res.* **2016**, *36*, 3833–3838.
22. Lee, C.; Siu, A.; Ramos, D.M. Multicellular spheroids as a model for hypoxia-induced EMT. *Anticancer Res.* **2016**, *36*, 6259–6263, doi:10.21873/anticanres.11220.
23. Lee, S.H.; Rigas, N.K.; Lee, C.R.; Bang, A.; Srikanth, S.; Gwack, Y.; Kang, M.K.; Kim, R.H.; Park, N.H.; Shin, K.H. Orai1 promotes tumor progression by enhancing cancer stemness via NFAT signaling in oral/oropharyngeal squamous cell carcinoma. *Oncotarget* **2016**, *7*, 43239–43255, doi:10.18632/oncotarget.9755.
24. Moon, Y.H.; Kim, D.; Sohn, H.M.; Lim, W. Effect of CD133 overexpression on the epithelial-to-mesenchymal transition in oral cancer cell lines. *Clinical and Experimental Metastasis* **2016**, *33*, 487–496, doi:10.1007/s10585-016-9793-y.
25. Patel, S.; Rawal, R. Role of miRNA dynamics and cytokine profile in governing CD44v6/Nanog/PTEN axis in oral cancer: Modulating the master regulators. *Tumor Biology* **2016**, *37*, 14565–14575, doi:10.1007/s13277-016-5289-2.
26. de Campos, P.S.; Matte, B.F.; Diel, L.F.; Jesus, L.H.; Bernardi, L.; Alves, A.M.; Rados, P.V.; Lamers, M.L. Low Doses of Curcuma longa Modulates Cell Migration and Cell–Cell Adhesion. *Phytother. Res.* **2017**, *31*, 1433–1440, doi:10.1002/ptr.5872.
27. Hagemann, J.; Jacobi, C.; Hahn, M.; Schmid, V.; Welz, C.; Schwenk-Zieger, S.; Stauber, R.; Baumeister, P.; Becker, S. Spheroid-based 3D Cell Cultures Enable Personalized Therapy Testing and Drug Discovery in Head and Neck Cancer. *Anticancer Res* **2017**, *37*, 2201–2210, doi:10.21873/anticanres.11555.
28. Leong, W.Y.; Soon, C.F.; Wong, S.C.; Tee, K.S.; Cheong, S.C.; Gan, S.H.; Youseffi, M. In vitro growth of human keratinocytes and oral cancer cells into microtissues: An aerosol-based microencapsulation technique. *Bioengineering* **2017**, *4*, doi:10.3390/bioengineering4020043.
29. Mendonca, B.D.S.; Agostini, M.; Aquino, I.G.; Dias, W.B.; Bastos, D.C.; Rumjanek, F.D. Suppression of MAGE-A10 alters the metastatic phenotype of tongue squamous cell carcinoma cells. *Biochem Biophys Rep* **2017**, *10*, 267–275, doi:S2405-5808(16)30348-X.
30. Mohanta, S.; Siddappa, G.; Valiyaveedan, S.G.; Ramanjanappa, R.D.T.; Das, D.; Pandian, R.; Khora, S.S.; Kuriakose, M.A.; Suresh, A. Cancer stem cell markers in patterning differentiation and in prognosis of oral squamous cell carcinoma. *Tumor Biology* **2017**, *39*, doi:10.1177/1010428317703656.
31. O'Rourke, C.; Hopper, C.; MacRobert, A.J.; Phillips, J.B.; Woodhams, J.H. Could clinical photochemical internalisation be optimised to avoid neuronal toxicity? *International Journal of Pharmaceutics* **2017**, *528*, 133–143, doi:10.1016/j.ijpharm.2017.05.071.
32. Ong, L.J.Y.; Islam, A.B.; DasGupta, R.; Iyer, N.G.; Leo, H.L.; Toh, Y.C. A 3D printed microfluidic perfusion device for multicellular spheroid cultures. *Biofabrication* **2017**, *9*, 12, doi:04500510.1088/1758-5090/aa8858.
33. Bano, N.; Yadav, M.; Das, B.C. Differential Inhibitory Effects of Curcumin Between HPV+ve and HPV-ve Oral Cancer Stem Cells. *Front. Oncol.* **2018**, *8*, 6, doi:41210.3389/fonc.2018.00412.
34. Essid, N.; Chambard, J.C.; Elgaaied, A.B. Induction of epithelial-mesenchymal transition (EMT) and Gli1 expression in head and neck squamous cell carcinoma (HNSCC) spheroid cultures. *Bosn J Basic Med Sci* **2018**, *18*, 336–346, doi:10.17305/bjbms.2018.3243.
35. Fujibayashi, E.; Yabuta, N.; Nishikawa, Y.; Uchihashi, T.; Miura, D.; Kurioka, K.; Tanaka, S.; Kogo, M.; Nojima, H. Isolation of cancer cells with augmented spheroid-forming capability using a novel tool equipped with removable filter. *Oncotarget* **2018**, *9*, 33931–33946, doi:10.18632/oncotarget.26092.
36. Guerreiro, E.M.; Vestad, B.; Steffensen, L.A.; Aass, H.C.D.; Saeed, M.; Øvstebø, R.; Costea, D.E.; Galtung, H.K.; Søland, T.M. Efficient extracellular vesicle isolation by combining cell media modifications, ultrafiltration, and size-exclusion chromatography. *PLoS ONE* **2018**, *13*, e0204276, doi:10.1371/journal.pone.0204276.

37. Hoornstra, D.; Vesterlin, J.; Parnanen, P.; Al-Samadi, A.; Zlotogorski-Hurvitz, A.; Vered, M.; Salo, T. Fermented lingonberry juice inhibits oral tongue squamous cell carcinoma invasion in vitro similarly to curcumin. *In Vivo* **2018**, *32*, 1089–1095, doi:10.21873/invivo.11350.
38. Lazarevic, M.; Milosevic, M.; Trisic, D.; Toljic, B.; Simonovic, J.; Nikolic, N.; Mikovic, N.; Jelovac, D.; Petrovic, M.; Vukadinovic, M.; et al. Putative cancer stem cells are present in surgical margins of oral squamous cell carcinoma. *Journal of B.U.ON.* **2018**, *23*, 1686–1692.
39. Noi, M.; Mukaisho, K.-I.; Yoshida, S.; Murakami, S.; Koshinuma, S.; Adachi, T.; Machida, Y.; Yamori, M.; Nakayama, T.; Yamamoto, G.; et al. ERK phosphorylation functions in invadopodia formation in tongue cancer cells in a novel silicate fibre-based 3D cell culture system. *International journal of oral science* **2018**, *10*, 30–30, doi:10.1038/s41368-018-0033-y.
40. Rivera, C.; Zandonadi, F.S.; Sánchez-Romero, C.; Soares, C.D.; Granato, D.C.; González-Arriagada, W.A.; Paes Leme, A.F. Agrin has a pathological role in the progression of oral cancer. *British Journal of Cancer* **2018**, *118*, 1628–1638, doi:10.1038/s41416-018-0135-5.
41. Roy, S.; Roy, S.; Kar, M.; Padhi, S.; Saha, A.; Anuja, K.; Banerjee, B. Role of p38 MAPK in disease relapse and therapeutic resistance by maintenance of cancer stem cells in head and neck squamous cell carcinoma. *J Oral Pathol Med* **2018**, *47*, 492–501, doi:10.1111/jop.12707.
42. Saha, A.; Roy, S.; Kar, M.; Roy, S.; Thakur, S.; Padhi, S.S.; Akhter, Y.; Banerjee, B. Role of Telomeric TRF2 in Orosphere Formation and CSC Phenotype Maintenance Through Efficient DNA Repair Pathway and its Correlation with Recurrence in OSCC. *Stem Cell Rev. Rep.* **2018**, *14*, 871–887, doi:10.1007/s12015-018-9823-z.
43. Sievers, D.; Bunzendahl, J.; Frosch, A.; Perske, C.; Hemmerlein, B.; Schliephake, H.; Brockmeyer, P. Generation of highly differentiated BHY oral squamous cell carcinoma multicellular spheroids. *Mol. Clin. Oncol.* **2018**, *8*, 323–325, doi:10.3892/mco.2017.1514.
44. Xie, S.L.; Fan, S.; Zhang, S.Y.; Chen, W.X.; Li, Q.X.; Pan, G.K.; Zhang, H.Q.; Wang, W.W.; Weng, B.; Zhang, Z.; et al. SOX8 regulates cancer stem-like properties and cisplatin-induced EMT in tongue squamous cell carcinoma by acting on the Wnt/beta-catenin pathway. *Int. J. Cancer* **2018**, *142*, 1252–1265, doi:10.1002/ijc.31134.
45. Almahmoudi, R.; Salem, A.; Murshid, S.; Dourado, M.R.; Apu, E.H.; Salo, T.; Al-Samadi, A. Interleukin-17F has anti-tumor effects in oral tongue cancer. *Cancers* **2019**, *11*, doi:10.3390/cancers11050650.
46. Chen, W.C.; Zou, A.M.; Zhang, H.Y.; Fu, X.Y.; Yao, F.; Yang, A.K. Odd-skipped related transcription factor 1 (OSR1) suppresses tongue squamous cell carcinoma migration and invasion through inhibiting NF-kappa B pathway. *Eur. J. Pharmacol.* **2018**, *839*, 33–39, doi:10.1016/j.ejphar.2018.09.020.
47. Furqan, M.; Huma, Z.; Ashfaq, Z.; Nasir, A.; Ullah, R.; Bilal, A.; Iqbal, M.; Khalid, M.H.; Hussain, I.; Faisal, A. Identification and evaluation of novel drug combinations of Aurora kinase inhibitor CCT137690 for enhanced efficacy in oral cancer cells. *Cell Cycle* **2019**, *18*, 2281–2292, doi:10.1080/15384101.2019.1643658.
48. Gawas, N.P.; Navarange, S.S.; Chovatiya, G.L.; Chaturvedi, P.; Waghmare, S.K. Establishment and characterization of novel human oral squamous cell carcinoma cell lines from advanced-stage tumors of buccal mucosa. *Oncology Reports* **2019**, *41*, 2289–2298, doi:10.3892/or.2019.7003.
49. Kochanek Stanton, J.; Close David, A.; Johnston Paul, A. High Content Screening Characterization of Head and Neck Squamous Cell Carcinoma Multicellular Tumor Spheroid Cultures Generated in 384-Well Ultra-Low Attachment Plates to Screen for Better Cancer Drug Leads. *ASSAY and Drug Development Technologies* **2019**, *17*, 17–36, doi:10.1089/adt.2018.896.
50. Lang, L.; Shay, C.; Zhao, X.; Xiong, Y.; Wang, X.; Teng, Y. Simultaneously inactivating Src and AKT by saracatinib/capivasertib co-delivery nanoparticles to improve the efficacy of anti-Src therapy in head and neck squamous cell carcinoma. *J. Hematol Oncol.* **2019**, *12*, 132, doi:10.1186/s13045-019-0827-1.
51. Roh, V.; Hiou-Feige, A.; Misetic, V.; Rivals, J.-P.; Sponarova, J.; Teh, M.-T.; Ferreira Lopes, S.; Truan, Z.; Mermod, M.; Monnier, Y.; et al. The transcription factor FOXM1 regulates the balance between proliferation and aberrant differentiation in head and neck squamous cell carcinoma. *The Journal of Pathology 0*, doi:10.1002/path.5342.
52. Su, Z.; Liu, D.; Chen, L.; Zhang, J.; Ru, L.; Chen, Z.; Gao, Z.; Wang, X. CD44-Targeted Magnetic Nanoparticles Kill Head And Neck Squamous Cell Carcinoma Stem Cells In An Alternating Magnetic Field. *International journal of nanomedicine* **2019**, *14*, 7549–7560, doi:10.2147/ijn.S215087.
53. Wessely, A.; Waltera, A.; Reichert, T.E.; Stockl, S.; Grassel, S.; Bauer, R.J. Induction of ALP and MMP9 activity facilitates invasive behavior in heterogeneous human BMSC and HNSCC 3D spheroids. *FASEB J.* **2019**, doi:10.1096/fj.201900925R.

Table S4. Studies utilizing organotypic raft cultures in OSCC.

Study No.	Authors (Year Published)	Type of Cell Used and Site from Which Cells Were Derived	Whether Co-Culture Used	Dermal Equivalent/Scaffold Used
1	Eicher and Lotan (1996) [1]	Cancer cell line (1483—retromolar trigone).	Yes—primary fibroblasts	Rat tail collagen type I
2	Eicher et al., (1996) [2]	Cancer cell line (TR146—buccal mucosa SCC metastasis to cervical lymph node).	Yes—primary fibroblasts	Rat tail collagen type I
3	Yoo et al., (2000) [3]	Normal oral keratinocyte cell line (IGHK—gingiva).<br>Cancer cell line (HN12—tongue).	Yes—sub-lethal irradiated NIH 3T3 fibroblasts as a feeder layer	Rat tail collagen type I
4	Hansson et al., (2001) [4]	Normal oral keratinocyte cell line (NOK—oral cavity).<br>Transformed (dysplastic) cell line (SVpgC2a—buccal mucosa).<br>Cancer cell line (SqCC/Y1—oral cavity).	Yes—primary fibroblasts	Rat tail collagen type I
5	Khan et al., (2001) [5]	Cancer cell lines (HSC2, HSC3, HSC4, OSC19, and OSC20—tongue SCC metastasis to cervical lymph node).	Yes—primary fibroblasts	Collagen type 1
6	Kinsen et al., (2003) [6]	Cancer cell lines (SAS—tongue, OSC-19—tongue SCC metastasis to cervical lymph node).	No	Collagen type 1
7	Costea et al., (2003) [7]	Primary oral keratinocytes.	Yes—primary oral fibroblasts	Collagen type 1
8	Costea et al., (2005) [8]	Normal oral keratinocyte cell line (NOK—oral cavity).<br>Dysplastic cell line (DOK—tongue).<br>Cancer cell line (PECA/PJ15—tongue).	Yes—primary oral fibroblast used as feeder layer	Collagen type 1
9	Lee et al., (2005) [9]	Normal oral keratinocyte cell line (IHOK—oral cavity).<br>Cancer cell lines (HN4, HN12—floor of the mouth).	Yes—primary gingival fibroblast	Collagen type 1
10	Nystrom et al., (2005) [10]	Cancer cell lines (H357, VB6, C1—tongue, CA1—oral cavity).	Yes—foreskin fibroblast cell line	Collagen type 1
11	Ylipalosaari et al., (2005) [11]	Cancer cell lines (VB6, C1—tongue).	Yes—primary fibroblast	Collagen type 1
12	Costea et al., (2006) [12]	Dysplastic cell line (DOK- tongue).	Yes—primary oral fibroblasts from human and rat sources	Collagen type 1

13	Lee et al., (2006) [13]	Cancer cell lines (HN4, HN12—oral cavity).	Yes—primary gingival fibroblast	Collagen type 1
14	Vigneswaran et al., (2006) [14]	Normal oral keratinocytes—gingiva.<br>Dysplastic cell lines (Leuk1and Leuk2—tongue).<br>Cancer cell lines (MDA686TU, MDA686LN—oropharynx, MDA1386TU, MDA1386LN—hypopharynx).	Yes—primary fibroblast	Collagen type 1
15	Daly et al., (2008) [15]	Cancer cell line (CA1—oral cavity).	Yes—primary fibroblast	Collagen type 1
16	Gaballah et al., (2008) [16]	Primary cells & cell lines from oral epithelial dysplasia and normal oral epithelium.	Yes—primary fibroblasts from dysplastic mucosa	Collagen
17	Nielsen et al., (2008) [17]	Cancer cell line (SCC-71—oral cavity)	Yes—primary fibroblast used for one model	Matrigel and collagen type 1
18	Husvik et al., (2009) [18]	Cancer cell lines (PE/CA-PJ15, PE/CA-PJ46, PE/CA-PJ49—tongue, PE/CA-PJ34, PE/CA-PJ41—oral cavity)	Yes—primary fibroblast	Collagen type 1
19	Kulasekara et al., (2009) [19]	Cancer cell line (PE/CA-PJ15—tongue).<br>Dysplastic cell line (DOK—tongue).<br>Normal oral keratinocyte—oral cavity.	Yes—normal oral fibroblasts used for one of the models	Collagen type 1
20	Yap et al., (2009) [20]	Cancer cell line (VB6—tongue).	Yes—fibroblast cell lines	50:50 mixture of Matrigel and collagen type 1
21	Nurmenniemi et al., (2009) [21]	Cancer cell line (HSC3—tongue).<br>Dysplastic cell line (DOK—tongue).	Yes—primary gingival fibroblasts	Collagen type 1 and human uterine leiomyoma discs
22	Brusevold et al., (2010) [22]	Cancer cell line (PE/CA-PJ49—tongue).	Yes—primary gingival fibroblasts	Rat tail collagen type I
23	Brusevold et al., (2010) [23]	Cancer cell line (PE/CA-PJ49—tongue).<br>Normal oral keratinocyte—oral cavity.	Yes—normal oral fibroblasts	Rat tail collagen type I
24	Colley et al., (2011) [24]	Cancer cell line (Cal27—tongue).<br>Dysplastic cell line (D20—tongue).<br>Primary normal oral keratinocytes—buccal mucosa and gingiva.	Yes—primary oral fibroblasts	De-cellularised and de-epidermised dermis

25	Shin et al., (2011) [25]	Cancer cell lines (SCC4, SCC-9, SCC-15– tongue, 1483—retromolar trigone).	Yes—normal oral fibroblasts	Collagen type 1
26	Aikio et al., (2012) [26]	Cancer cell line (HSC-3—tongue SCC metastasized to cervical lymph node).	Yes—primary gingival fibroblasts	Collagen type 1
27	Campisi et al., (2012) [27]	Cancer cell line (SCC4—tongue).	No	Matrigel
28	Chaw et al., (2012) [28]	Cancer cell line (PE/CA-PJ15—tongue).	No	Acellular de-epidermized dermis
29	Khan et al., (2012) [29]	Cancer cell line (H400—oral cavity).	Yes—3T3 irradiated fibroblasts used as a feeder layer	Acellular de-epidermized dermis and collagen
30	Rautava et al., (2012) [30]	Immortalised gingival keratinocytes.	Yes—immortalised gingival fibroblasts	Acellular de-epidermized dermis
31	Vilen et al., (2012) [31]	Cancer cell lines (HSC-3—tongue SCC metastasized to cervical lymph node).	No	Human uterine leiomyoma discs
32	AbdulMajeed et al., (2013) [32]	Cancer cell line (PE/CA-PJ15—tongue).<br>Dysplastic cell lines (DOK, PoE-9n—tongue).<br>Normal oral keratinocyte cell line (OKF6-TERT2—oral cavity).	No	Acellular de-epidermized dermis
33	Bitu et al., (2013) [33]	Cancer cell line (HSC-3—tongue SCC metastasized to cervical lymph node).	Yes—primary gingival fibroblasts	Rat tail collagen type I
34	Dalley et al., (2013) [34]	Cancer cell line (PE/CA-PJ15—tongue).<br>Dysplastic cell lines (DOK, PoE-9n—tongue).<br>Normal oral keratinocyte cell line (OKF6-TERT2—oral cavity).	No	Acellular de-epidermized dermis
35	Salo et al., (2013) [35]	Cancer cell lines (HSC-3—tongue SCC metastasized to cervical lymph node, SAS- tongue)	Yes—bone marrow-derived multipotent mesenchymal stromal cells	Human uterine leiomyoma discs
36	Scanlon et al., (2013) [36]	Cancer cell line (UM-SCC-1—floor of mouth).	No	AlloDerm (LifeCell)—an acellular dermal matrix and human type IV collagen
37	Teppo et al., (2013) [37]	Cancer cell lines (HSC-3—tongue SCC metastasized to cervical lymph node, UT-SCC-43A—primary carcinoma of mandibular gingiva,<br>UT-SCC-43B- recurrent carcinoma of mandibular gingiva).	No	Human uterine leiomyoma discs

38	van Tubergen et al., (2013) [38]	Cancer cell Line—oropharynx. Normal oral keratinocyte cell line—oral cavity.	No	AlloDerm (LifeCell)—an acellular dermal matrix and human type IV collagen
39	Brusevold et al., (2014) [39]	Cancer cell lines (PE/CA-PJ-49—tongue, PE/CA-PJ-41—oral squamous epithelium, SCC-9—tongue).	Yes—normal oral fibroblasts	Collagen
40	Alahuhta et al., (2015) [40]	Cancer cell line (HSC-3—tongue SCC metastasized to cervical lymph node).	Yes—primary fibroblasts	Rat tail collagen type I
41	Kauppila et al., (2015) [41]	Cancer cell lines (HSC-3—tongue SCC metastasized to cervical lymph node, SCC25, SCC15—tongue).	No	Human uterine leiomyoma discs
42	Omar et al., (2015) [42]	Cancer cell lines (HSC-3—tongue SCC metastasized to cervical lymph node, SAS- tongue).	No	Human uterine leiomyoma discs
43	Pirila et al., (2015) [43]	Cancer cell line (HSC-3—tongue SCC metastasized to cervical lymph node).	Yes—leukaemia cells	Human uterine leiomyoma discs
44	Sapkota et al., (2015) [44]	Cancer cell line (H357—tongue).	Yes—primary carcinoma-associated fibroblasts	Collagen type 1
45	Vered et al., (2015) [45]	Cancer cell line (HSC-3—tongue SCC metastasized to cervical lymph node).	Yes—primary fibroblasts obtained from the vicinity of a tongue SCC	Human uterine leiomyoma discs
46	Buskermolen et al., (2016) [46]	Primary cells—gingival keratinocytes.	Yes—primary human gingival fibroblasts	Collagen
47	Hwang et al., (2016) [47]	Cancer cell line (YD-10B—tongue).	Yes—primary human gingival fibroblasts	Collagen type 1A
48	Makinen et al., (2016) [48]	Cancer cell lines (HSC-3—tongue SCC metastasized to cervical lymph node).	No	Human uterine leiomyoma discs
49	Sawant et al., (2016) [49]	Primary cells—normal & dysplastic oral keratinocytes and OSCC cells from the tongue.	Yes—primary human tongue fibroblasts	Collagen
50	Simonik et al., (2016) [50]	Cancer cell line (VU-SCC-1729—oral cavity).	Yes—oesophageal fibroblasts	3:1 collagen type I and Matrigel
51	Sundquist et al., (2016) [51]	Cancer cell lines (HSC-3—tongue SCC metastasized to cervical lymph node, SCC4, SCC15—tongue).	No	Human uterine leiomyoma discs

52	Zanetti et al., (2016) [52]	Commercially available oral epithelial cell culture model—EpiOral (ORL-200)™.	No	Collagen
53	Al-Samadi et al., (2017) [53]	Cancer cell lines (HSC-3—tongue SCC metastasized to cervical lymph node, SCC 25—tongue).	Yes—peripheral blood monocytes	Human uterine leiomyoma discs
54	Koravala et al., (2017) [54]	Cancer cell lines (HSC-3—tongue SCC metastasized to cervical lymph node, SCC15—tongue).	No	Human uterine leiomyoma discs
55	Moilanen et al., (2017) [55]	Cancer cell lines (HSC-3—tongue SCC metastasized to cervical lymph node, SCC25—tongue).	No	Human uterine leiomyoma discs
56	Almela et al., (2018) [56]	Primary cells—osteoblasts and oral keratinocytes. Cancer cell line (UPCI-SCC-090—Tongue).	Yes—primary oral fibroblasts for oral mucosa model	Rat tail collagen type I for oral mucosa model. Bioprinted bone scaffold for bone model.
57	Hoque Apu et al., (2018) [57]	Cancer cell line (SqCC/Y1- buccal mucosa).	No	Matrigel, rat tail collagen type 1, Human uterine leiomyoma discs
58	Väyrynen et al., (2019) [58]	Cancer cell line (HSC-3—tongue SCC metastasized to cervical lymph node).	No	Human uterine leiomyoma discs
59	Dourado et al., (2019) [59]	Cancer cell lines (HSC-3—tongue SCC metastasized to cervical lymph node, SAS, SCC-15 and SCC-25—tongue).	Yes—normal oral and cancer-associated fibroblasts	Human uterine leiomyoma discs
60	Iwai et al., (2019) [60]	Cancer cell lines (HSC-3—tongue SCC metastasized to cervical lymph node, SAS—tongue).	Yes- primary dermal fibroblasts and lymphatic endothelial cells	3D tissue constructs consisted of 5 layers of fibroblast, 1 layer of endothelial cells and five layers of fibroblasts. All cells were coated with fibronectin and laminin.
61	Lee et al., (2019) [61]	Cancer cell line (HSC-3—tongue SCC metastasized to cervical lymph node).	No	Collagen type 1A
62	Väyrynen et al., (2019) [62]	Cancer cell lines (HSC-3—tongue SCC metastasized to cervical lymph node, SAS, SCC-15 and SCC-25—tongue).	No	Human uterine leiomyoma discs

References

1. Eicher, S.A.; Lotan, R. Differential effects of retinoic acid and N-(4-hydroxyphenyl)retinamide on head and neck squamous cell carcinoma cells. *Laryngoscope* **1996**, *106*, 1471–1475, doi:10.1097/00005537-199612000-00005.
2. Eicher, S.A.; Clayman, G.L.; Liu, T.J.; Shillitoe, E.J.; Storthz, K.A.; Roth, J.A.; Lotan, R. Evaluation of topical gene therapy for head and neck squamous cell carcinoma in an organotypic model. *Clinical Cancer Research* **1996**, *2*, 1659–1664.
3. Yoo, G.H.; Washington, J.; Piechocki, M.; Ensley, J.; Shibuya, T.; Oda, D.; Wei, W.Z. Progression of head and neck cancer in an in vitro model. *Archives of Otolaryngology-Head and Neck Surgery* **2000**, *126*, 1313–1318, doi:10.1001/archotol.126.11.1313.
4. Hansson, A.; Bloor, B.K.; Haig, Y.; Morgan, P.R.; Ekstrand, J.; Grafström, R.C. Expression of keratins in normal, immortalized and malignant oral epithelia in organotypic culture. *Oral Oncology* **2001**, *37*, 419–430, doi:10.1016/s1368-8375(00)00089-0.
5. Khan, M.H.; Yasuda, M.; Higashino, F.; Haque, S.; Kohgo, T.; Nakamura, M.; Shindoh, M. nm23-H1 suppresses invasion of oral squamous cell carcinoma-derived cell lines without modifying matrix metalloproteinase-2 and matrix metalloproteinase-9 expression. *Am. J. Pathol.* **2001**, *158*, 1785–1791, doi:10.1016/s0002-9440(10)64134-x.
6. Kinsenn, H.; Sato, H.; Furukawa, M.; Yoshizaki, T. Modulation of cell growth and matrix metalloproteinase-2 activation of oral squamous cell carcinoma as a function of culture condition with type I collagen. *Acta Otolaryngol* **2003**, *123*, 987–993, doi:10.1080/00016480310016109 [doi].
7. Costea, D.E.; Loro, L.L.; Dimba, E.A.O.; Vintermyr, O.K.; Johannessen, A.C. Crucial effects of fibroblasts and keratinocyte growth factor on morphogenesis of reconstituted human oral epithelium. *J. Invest. Dermatol.* **2003**, *121*, 1479–1486, doi:10.1111/j.1523-1747.2003.12616.x.
8. Costea, D.E.; Johannessen, A.C.; Vintermyr, O.K. Fibroblast control on epithelial differentiation is gradually lost during in vitro tumor progression. *Differentiation* **2005**, *73*, 134–141, doi:10.1111/j.1432-0436.2005.00017.x.
9. Lee, H.J.; Guo, H.Y.; Lee, S.K.; Jeon, B.H.; Jun, C.D.; Lee, S.K.; Park, M.H.; Kim, E.C. Effects of nicotine on proliferation, cell cycle, and differentiation in immortalized and malignant oral keratinocytes. *Journal of Oral Pathology and Medicine* **2005**, *34*, 436–443, doi:10.1111/j.1600-0714.2005.00342.x.
10. Nystrom, M.L.; Thomas, G.J.; Stone, M.; Mackenzie, I.C.; Hart, I.R.; Marshall, J.F. Development of a quantitative method to analyse tumour cell invasion in organotypic culture. *J Pathol* **2005**, *205*, 468–475, doi:10.1002/path.1716 [doi].
11. Ylipalosaari, M.; Thomas, G.J.; Nystrom, M.; Salhimi, S.; Marshall, J.F.; Huotari, V.; Tervahartiala, T.; Sorsa, T.; Salo, T. alpha v beta 6 integrin down-regulates the MMP-13 expression in oral squamous cell carcinoma cells. *Experimental Cell Research* **2005**, *309*, 273–283, doi:10.1016/j.yexcr.2005.06.008.
12. Costea, D.E.; Kulasekara, K.; Neppelberg, E.; Johannessen, A.C.; Vintermyr, O.K. Species-specific fibroblasts required for triggering invasiveness of partially transformed oral keratinocytes. *Am. J. Pathol.* **2006**, *168*, 1889–1897, doi:10.2353/ajpath.2006.050843.
13. Lee, H.J.; Son, D.H.; Lee, S.K.; Lee, J.; Jun, C.D.; Jeon, B.H.; Lee, S.K.; Kim, E.C. Extract of Coptidis rhizoma induces cytochrome-c dependent apoptosis in immortalized and malignant human oral keratinocytes. *Phytother. Res.* **2006**, *20*, 773–779, doi:10.1002/ptr.1956.
14. Vigneswaran, N.; Beckers, S.; Waigel, S.; Mensah, J.; Wu, J.; Mo, J.; Fleisher, K.E.; Bouquot, J.; Sacks, P.G.; Zacharias, W. Increased EMMPRIN (CD 147) expression during oral carcinogenesis. *Experimental and Molecular Pathology* **2006**, *80*, 147–159, doi:10.1016/j.yexmp.2005.09.011.
15. Daly, A.J.; McIlreavey, L.; Irwin, C.R. Regulation of HGF and SDF-1 expression by oral fibroblasts-Implications for invasion of oral cancer. *Oral Oncology* **2008**, *44*, 646–651, doi:10.1016/j.oraloncology.2007.08.012.
16. Gaballah, K.; Costea, D.E.; Hills, A.; Gollin, S.M.; Harrison, P.; Partridge, M. Tissue engineering of oral dysplasia. *J. Pathol.* **2008**, *215*, 280–289, doi:10.1002/path.2360.
17. Nielsen, J.D.; Moeslund, M.; Wandall, H.H.; Dabelsteen, S. Influences of tumor stroma on the malignant phenotype. *Journal of Oral Pathology and Medicine* **2008**, *37*, 412–416, doi:10.1111/j.1600-0714.2008.00655.x.
18. Husvik, C.; Khuu, C.; Bryne, M.; Halstensen, T.S. PGE2 production in oral cancer cell lines is COX-2-dependent. *J. Dent. Res.* **2009**, *88*, 164–169, doi:10.1177/0022034508329519.

19. Kulasekara, K.K.; Lukandu, O.M.; Neppelberg, E.; Vintermyr, O.K.; Johannessen, A.C.; Costea, D.E. Cancer progression is associated with increased expression of basement membrane proteins in three-dimensional in vitro models of human oral cancer. *Archives of Oral Biology* **2009**, *54*, 924–931, doi:10.1016/j.archoralbio.2009.07.004.
20. Yap, L.F.; Jenei, V.; Robinson, C.M.; Moutasim, K.; Benn, T.M.; Threadgold, S.P.; Lopes, V.; Wei, W.; Thomas, G.J.; Paterson, I.C. Upregulation of Eps8 in oral squamous cell carcinoma promotes cell migration and invasion through integrin-dependent Rac1 activation. *Oncogene* **2009**, *28*, 2524–2534, doi:10.1038/onc.2009.105.
21. Nurmenniemi, S.; Sinikumpu, T.; Alahuhta, I.; Salo, S.; Sutinen, M.; Santala, M.; Risteli, J.; Nyberg, P.; Salo, T. A novel organotypic model mimics the tumor microenvironment. *Am. J. Pathol.* **2009**, *175*, 1281–1291, doi:10.2353/ajpath.2009.081110.
22. Brusevold, I.J.; Husvik, C.; Schreurs, O.; Schenck, K.; Bryne, M.; Soland, T.M. Induction of invasion in an organotypic oral cancer model by CoCl2, a hypoxia mimetic. *Eur. J. Oral Sci.* **2010**, *118*, 168–176, doi:10.1111/j.1600-0722.2010.00720.x.
23. Brusevold, I.J.; Søland, T.M.; Khuu, C.; Christoffersen, T.; Bryne, M. Nuclear and cytoplasmic expression of Met in oral squamous cell carcinoma and in an organotypic oral cancer model. *Eur. J. Oral Sci.* **2010**, *118*, 342–349, doi:10.1111/j.1600-0722.2010.00747.x.
24. Colley, H.E.; Hearnden, V.; Jones, A.V.; Weinreb, P.H.; Violette, S.M.; MacNeil, S.; Thornhill, M.H.; Murdoch, C. Development of tissue-engineered models of oral dysplasia and early invasive oral squamous cell carcinoma. *British Journal of Cancer* **2011**, *105*, 1582–1592, doi:10.1038/bjc.2011.403.
25. Shin, K.H.; Bae, S.D.; Hong, H.S.; Kim, R.H.; Kang, M.K.; Park, N.H. MiR-181a shows tumor suppressive effect against oral squamous cell carcinoma cells by downregulating K-ras. *Biochemical and Biophysical Research Communications* **2011**, *404*, 896–902, doi:10.1016/j.bbrc.2010.12.055.
26. Aikio, M.; Alahuhta, I.; Nurmenniemi, S.; Suojanen, J.; Palovuori, R.; Teppo, S.; Sorsa, T.; López-Otín, C.; Pihlajaniemi, T.; Salo, T.; et al. Arresten, a Collagen-Derived Angiogenesis Inhibitor, Suppresses Invasion of Squamous Cell Carcinoma. *PLoS ONE* **2012**, *7*, doi:10.1371/journal.pone.0051044.
27. Campisi, G.; Giannola, L.I.; Fucarino, A.; Marino Gammazza, A.; Pitruzzella, A.; Marciano, V.; De Caro, V.; Siragusa, M.G.; Giandalia, G.; Compilato, D.; et al. Medium-term culture of primary oral squamous cell carcinoma in a three- dimensional model: Effects on cell survival following topical 5-fluororacile delivery by drug-loaded matrix tablets. *Curr Pharm Des* **2012**, *18*, 5411–5420, doi:CPD-EPUB-20120525-4 [pii]10.2174/138161212803307536 [doi].
28. Chaw, S.Y.; Abdul Majeed, A.; Dalley, A.J.; Chan, A.; Stein, S.; Farah, C.S. Epithelial to mesenchymal transition (EMT) biomarkers–E-cadherin, beta-catenin, APC and Vimentin–in oral squamous cell carcinogenesis and transformation. *Oral Oncology* **2012**, *48*, 997–1006, doi:10.1016/j.oraloncology.2012.05.011.
29. Khan, E.; Shelton, R.M.; Cooper, P.R.; Hamburger, J.; Landini, G. Architectural characterization of organotypic cultures of H400 and primary rat keratinocytes. *J. Biomed. Mater. Res. Part A* **2012**, *100A*, 3227-3238, doi:10.1002/jbm.a.34263.
30. Rautava, J.; Pollanen, M.; Laine, M.A.; Willberg, J.; Lukkarinen, H.; Soukka, T. Effects of tacrolimus on an organotypic raft-culture model mimicking oral mucosa. *Clin. Exp. Dermatol.* **2012**, *37*, 897–903, doi:10.1111/j.1365-2230.2012.04372.x.
31. Vilen, S.T.; Suojanen, J.; Salas, F.; Risteli, J.; Ylipalosaari, M.; Itkonen, O.; Koistinen, H.; Baumann, M.; Stenman, U.H.; Sorsa, T.; et al. Trypsin-2 enhances carcinoma invasion by processing tight junctions and activating promt1-mmp. *Cancer Invest.* **2012**, *30*, 583–592, doi:10.3109/07357907.2012.716467.
32. Abdulmajeed, A.A.; Dalley, A.J.; Farah, C.S. Loss of ELF3 immunoexpression is useful for detecting oral squamous cell carcinoma but not for distinguishing between grades of epithelial dysplasia. *Annals of Diagnostic Pathology* **2013**, *17*, 331–340, doi:10.1016/j.anndiagpath.2013.03.003.
33. Bitu, C.C.; Kauppila, J.H.; Bufalino, A.; Nurmenniemi, S.; Teppo, S.; Keinänen, M.; Vilen, S.-T.; Lehenkari, P.; Nyberg, P.; Coletta, R.D.; et al. Cathepsin K is present in invasive oral tongue squamous cell carcinoma in vivo and in vitro. *PLoS ONE* **2013**, *8*, e70925–e70925, doi:10.1371/journal.pone.0070925.
34. Dalley, A.J.; Abdulmajeed, A.A.; Upton, Z.; Farah, C.S. Organotypic culture of normal, dysplastic and squamous cell carcinoma-derived oral cell lines reveals loss of spatial regulation of CD44 and p75NTR in malignancy. *Journal of Oral Pathology and Medicine* **2013**, *42*, 37–46, doi:10.1111/j.1600-0714.2012.01170.x.

35. Salo, S.; Bitu, C.; Merkku, K.; Nyberg, P.; Bello, I.O.; Vuoristo, J.; Sutinen, M.; Vahanikkila, H.; Costea, D.E.; Kauppila, J.H.; et al. Human bone marrow mesenchymal stem cells induce collagen production and tongue cancer invasion. *PLoS ONE* **2013**, *8*, e77692, doi:10.1371/journal.pone.0077692 [doi]PONE-D-13-24917 [pii].
36. Scanlon, C.S.; Van Tubergen, E.A.; Chen, L.-C.; Elahi, S.F.; Kuo, S.; Feinberg, S.; Mycek, M.-A.; D'Silva, N.J. Characterization of squamous cell carcinoma in an organotypic culture via subsurface non-linear optical molecular imaging. *Experimental biology and medicine (Maywood, N.J.)* **2013**, *238*, 1233–1241, doi:10.1177/1535370213502628.
37. Teppo, S.; Sundquist, E.; Vered, M.; Holappa, H.; Parkkisenniemi, J.; Rinaldi, T.; Lehenkari, P.; Grenman, R.; Dayan, D.; Risteli, J.; et al. The hypoxic tumor microenvironment regulates invasion of aggressive oral carcinoma cells. *Experimental Cell Research* **2013**, *319*, 376–389, doi:10.1016/j.yexcr.2012.12.010.
38. Van Tubergen, E.A.; Banerjee, R.; Liu, M.; Vander Broek, R.; Light, E.; Kuo, S.; Feinberg, S.E.; Willis, A.L.; Wolf, G.; Carey, T.; et al. Inactivation or loss of TTP promotes invasion in head and neck cancer via transcript stabilization and secretion of MMP9, MMP2, and IL-6. *Clin Cancer Res* **2013**, *19*, 1169–1179, doi:1078-0432.CCR-12-2927 [pii]10.1158/1078-0432.CCR-12-2927 [doi].
39. Brusevold, I.J.; Tveteraas, I.H.; Aasrum, M.; Ødegård, J.; Sandnes, D.L.; Christoffersen, T. Role of LPAR3, PKC and EGFR in LPA-induced cell migration in oral squamous carcinoma cells. *BMC Cancer* **2014**, *14*, doi:10.1186/1471-2407-14-432.
40. Alahuhta, I.; Aikio, M.; Väyrynen, O.; Nurmenniemi, S.; Suojanen, J.; Teppo, S.; Pihlajaniemi, T.; Heljasvaara, R.; Salo, T.; Nyberg, P. Endostatin induces proliferation of oral carcinoma cells but its effect on invasion is modified by the tumor microenvironment. *Experimental Cell Research* **2015**, *336*, 130–140, doi:10.1016/j.yexcr.2015.06.012.
41. Kauppila, J.H.; Korvala, J.; Siirilä, K.; Manni, M.; Mäkinen, L.K.; Hagström, J.; Atula, T.; Haglund, C.; Selander, K.S.; Saarnio, J.; et al. Toll-like receptor 9 mediates invasion and predicts prognosis in squamous cell carcinoma of the mobile tongue. *Journal of Oral Pathology and Medicine* **2015**, *44*, 571–577, doi:10.1111/jop.12272.
42. Omar, A.A.H.; Korvala, J.; Haglund, C.; Virolainen, S.; Hayry, V.; Atula, T.; Kontio, R.; Rihtniemi, J.; Pihakari, A.; Sorsa, T.; et al. Toll-like receptors-4 and-5 in oral and cutaneous squamous cell carcinomas. *Journal of Oral Pathology & Medicine* **2015**, *44*, 258–265, doi:10.1111/jop.12233.
43. Pirila, E.; Vayrynen, O.; Sundquist, E.; Pakkila, K.; Nyberg, P.; Nurmenniemi, S.; Paakkonen, V.; Pesonen, P.; Dayan, D.; Vered, M.; et al. Macrophages modulate migration and invasion of human tongue squamous cell carcinoma. *PLoS ONE* **2015**, *10*, e0120895, doi:10.1371/journal.pone.0120895 [doi]PONE-D-14-50316 [pii].
44. Sapkota, D.; Bruland, O.; Parajuli, H.; Osman, T.A.; Teh, M.T.; Johannessen, A.C.; Costea, D.E. S100A16 promotes differentiation and contributes to a less aggressive tumor phenotype in oral squamous cell carcinoma. *BMC Cancer* **2015**, *15*, doi:10.1186/s12885-015-1622-1.
45. Vered, M.; Lehtonen, M.; Hotakainen, L.; Pirila, E.; Teppo, S.; Nyberg, P.; Sormunen, R.; Zlotogorski-Hurvitz, A.; Salo, T.; Dayan, D. Caveolin-1 accumulation in the tongue cancer tumor microenvironment is significantly associated with poor prognosis: An in-vivo and in-vitro study. *BMC Cancer* **2015**, *15*, 25, doi:10.1186/s12885-015-1030-6 [doi]s12885-015-1030-6 [pii].
46. Buskermolen, J.K.; Reijnders, C.M.A.; Spiekstra, S.W.; Steinberg, T.; Kleverlaan, C.J.; Feilzer, A.J.; Bakker, A.D.; Gibbs, S. Development of a Full-Thickness Human Gingiva Equivalent Constructed from Immortalized Keratinocytes and Fibroblasts. *Tissue Eng. Part C-Methods* **2016**, *22*, 781–791, doi:10.1089/ten.tec.2016.0066.
47. Hwang, Y.S.; Ahn, S.Y.; Moon, S.; Zheng, Z.; Cha, I.-H.; Kim, J.; Zhang, X. Insulin-like growth factor-II mRNA binding protein-3 and podoplanin expression are associated with bone invasion and prognosis in oral squamous cell carcinoma. *Archives of Oral Biology* **2016**, *69*, 25–32, doi:10.1016/j.archoralbio.2016.05.008.
48. Mäkinen, L.K.; Ahmed, A.; Hagström, J.; Lehtonen, S.; Mäkitie, A.A.; Salo, T.; Haglund, C.; Atula, T. Toll-like receptors 2, 4, and 9 in primary, metastasized, and recurrent oral tongue squamous cell carcinomas. *Journal of Oral Pathology and Medicine* **2016**, *45*, 338–345, doi:10.1111/jop.12373.
49. Sawant, S.; Dongre, H.; Singh, A.K.; Joshi, S.; Costea, D.E.; Mahadik, S.; Ahire, C.; Makani, V.; Dange, P.; Sharma, S.; et al. Establishment of 3D Co-Culture Models from Different Stages of Human Tongue Tumorigenesis: Utility in Understanding Neoplastic Progression. *PLoS ONE* **2016**, *11*, 20, doi:e016061510.1371/journal.pone.0160615.
50. Simonik, E.A.; Cai, Y.; Kimmelshue, K.N.; Brantley-Sieders, D.M.; Loomans, H.A.; Andl, C.D.; Westlake, G.M.; Youngblood, V.M.; Chen, J.; Yarbrough, W.G.; et al. LIM-Only Protein 4 (LMO4) and LIM Domain

Binding Protein 1 (LDB1) Promote Growth and Metastasis of Human Head and Neck Cancer (LMO4 and LDB1 in Head and Neck Cancer). *PLoS ONE* **2016**, *11*, e0164804, doi:10.1371/journal.pone.0164804 [doi]PONE-D-16-16733 [pii].

51. Sundquist, E.; Renko, O.; Salo, S.; Magga, J.; Cervigne, N.K.; Nyberg, P.; Risteli, J.; Sormunen, R.; Vuolteenaho, O.; Zandonadi, F.; et al. Neoplastic extracellular matrix environment promotes cancer invasion in vitro. *Experimental Cell Research* **2016**, *344*, 229–240, doi:10.1016/j.yexcr.2016.04.003.
52. Zanetti, F.; Sewer, A.; Mathis, C.; Iskandar, A.R.; Kostadinova, R.; Schlage, W.K.; Leroy, P.; Majeed, S.; Guedj, E.; Trivedi, K.; et al. Systems Toxicology Assessment of the Biological Impact of a Candidate Modified Risk Tobacco Product on Human Organotypic Oral Epithelial Cultures. *Chem. Res. Toxicol.* **2016**, *29*, 1252–1269, doi:10.1021/acs.chemrestox.6b00174.
53. Al-Samadi, A.; Awad, S.A.; Tuomainen, K.; Zhao, Y.; Salem, A.; Parikka, M.; Salo, T. Crosstalk between tongue carcinoma cells, extracellular vesicles, and immune cells in in vitro and in vivo models. *Oncotarget* **2017**, *8*, 60123–60134, doi:10.18632/oncotarget.17768.
54. Korvala, J.; Jee, K.; Porkola, E.; Almangush, A.; Mosakhani, N.; Bitu, C.; Cervigne, N.K.; Zandonadi, F.S.; Meirelles, G.V.; Leme, A.F.P.; et al. MicroRNA and protein profiles in invasive versus non-invasive oral tongue squamous cell carcinoma cells in vitro. *Experimental Cell Research* **2017**, *350*, 9–18, doi:10.1016/j.yexcr.2016.10.015.
55. Moilanen, J.M.; Loffek, S.; Kokkonen, N.; Salo, S.; Vayrynen, J.P.; Hurskainen, T.; Manninen, A.; Riihila, P.; Heljasvaara, R.; Franzke, C.W.; et al. Significant Role of Collagen XVII And Integrin beta4 in Migration and Invasion of The Less Aggressive Squamous Cell Carcinoma Cells. *Sci Rep* **2017**, *7*, 45057, doi:srep45057 [pii]10.1038/srep45057 [doi].
56. Almela, T.; Al-Sahaf, S.; Brook, I.M.; Khoshroo, K.; Rasoulianboroujeni, M.; Fahimipour, F.; Tahriri, M.; Dashtimoghadam, E.; Bolt, R.; Tayebi, L.; et al. 3D printed tissue engineered model for bone invasion of oral cancer. *Tissue and Cell* **2018**, *52*, 71–77, doi:10.1016/j.tice.2018.03.009.
57. Hoque Apu, E.; Akram, S.U.; Rissanen, J.; Wan, H.; Salo, T. Desmoglein 3—Influence on oral carcinoma cell migration and invasion. *Experimental Cell Research* **2018**, *370*, 353–364, doi:10.1016/j.yexcr.2018.06.037.
58. Väyrynen, O.; Piippo, M.; Jämsä, H.; Väisänen, T.; de Almeida, C.E.B.; Salo, T.; Missailidis, S.; Risteli, M. Effects of ionizing radiation and HPSE1 inhibition on the invasion of oral tongue carcinoma cells on human extracellular matrices in vitro. *Experimental Cell Research* **2018**, *371*, 151–161, doi:10.1016/j.yexcr.2018.08.005.
59. Dourado, M.R.; Korvala, J.; Åström, P.; De Oliveira, C.E.; Cervigne, N.K.; Mofatto, L.S.; Campanella Bastos, D.; Pereira Messetti, A.C.; Graner, E.; Paes Leme, A.F.; et al. Extracellular vesicles derived from cancer-associated fibroblasts induce the migration and invasion of oral squamous cell carcinoma. *Journal of Extracellular Vesicles* **2019**, *8*, doi:10.1080/20013078.2019.1578525.
60. Iwai, S.; Kishimoto, S.; Amano, Y.; Nishiguchi, A.; Matsusaki, M.; Takeshita, A.; Akashi, M. Three-dimensional cultured tissue constructs that imitate human living tissue organization for analysis of tumor cell invasion. *J Biomed Mater Res A* **2019**, *107*, 292–300, doi:10.1002/jbm.a.36319.
61. Lee, J.; Nho, Y.H.; Yun, S.K.; Hwang, Y.S. Anti-invasive and Anti-tumor Effects of Dryopteris crassirhizoma Extract by Disturbing Actin Polymerization. *Integr Cancer Ther* **2019**, *18*, 1534735419851197, doi:10.1177/1534735419851197.
62. Väyrynen, O.; Åström, P.; Nyberg, P.; Alahuhta, I.; Pirilä, E.; Vilen, S.-T.; Aikio, M.; Heljasvaara, R.; Risteli, M.; Sutinen, M.; et al. Matrix metalloproteinase 9 inhibits the motility of highly aggressive HSC-3 oral squamous cell carcinoma cells. *Experimental Cell Research* **2019**, *376*, 18–26, doi:10.1016/j.yexcr.2019.01.018.
